# Value of supplemental interventions to enhance the effectiveness of physical exercise during respiratory rehabilitation in COPD patients. A Systematic Review

**DOI:** 10.1186/1465-9921-5-25

**Published:** 2004-12-02

**Authors:** Milo A Puhan, Holger J Schünemann, Martin Frey, Lucas M Bachmann

**Affiliations:** 1 University of Zurich, Horten Centre, Switzerland; 2 University at Buffalo, Departments of Medicine and of Social & Preventive Medicine, New York, USA; 3 McMaster University, Department of Clinical Epidemiology and Biostatistics, Hamilton, Ontario, Canada; 4 Klinik Barmelweid, Department of Respiratory Medicine, Barmelweid, Switzerland; 5 University of Berne, Department of Social and Preventive Medicine, Berne, Switzerland

## Abstract

**Background:**

There is a controversy about the additional benefit of various supplemental interventions used in clinical practice to further enhance the effectiveness of respiratory rehabilitation in patients with Chronic obstructive pulmonary disease (COPD). The aim of this research was to assess randomised controlled trials (RCTs) testing the additional benefit of supplemental interventions during respiratory rehabilitation in COPD patients.

**Methods:**

Systematic review with literature searches in six electronic databases, extensive hand-searching and contacting of authors. Two reviewers selected independently eligible RCTs, rated the methodological quality and extracted the data, which were analyzed considering the minimal important difference of patient-important outcomes where possible.

**Findings:**

We identified 20 RCTs whereof 18 provided sufficient data for analysis. The methodological quality was low and sample sizes were too small for most trials to produce meaningful results (median total sample size = 28). Data from five trials showed that supplemental oxygen during exercise did not have clinically meaningful effects on health-related quality of life while improvements of exercise capacity may be even larger for patients exercising on room air. RCTs of adding assisted ventilation, nutritional supplements or a number of anabolically acting drugs do not provide sufficient evidence for or against the use any of these supplemental interventions.

**Interpretation:**

There is insufficient evidence for most supplemental interventions during respiratory rehabilitation to estimate their additional value, partly due to methodological shortcomings of included RCTs. Current data do not suggest benefit from supplemental oxygen during exercise, although the methodological quality of included trials limits conclusions. To appropriately assess any of the various supplemental interventions used in clinical practice, pragmatic trials on respiratory rehabilitation of COPD patients need to consider methodological aspects as well as appropriate sample sizes.

## Introduction

Chronic obstructive pulmonary disease (COPD) has a large impact on health-related quality of life (HRQL) and represents a major health burden in industrialized and developing countries [1-4]. A systematic review including 23 randomized controlled trials (RCTs) has shown that patients with COPD improve their HRQL and exercise capacity during respiratory rehabilitation[5]. Recent data on long-term outcomes after respiratory rehabilitation show reductions of exacerbations and hospitalizations [6-8].

Physical exercise is the central component of respiratory rehabilitation programs because it reverses peripheral muscle dysfunction[9], a highly prevalent comorbidity of COPD associated with increased risk of exacerbations and mortality[10,11]. While respiratory rehabilitation including physical exercise has become a cornerstone of COPD management [12-14], there is controversy about the additional value of several supplemental interventions to support exercise programs such as oxygen during exercise[15] or anabolically acting hormones[16].

Clinicians, who consider these supplemental interventions during respiratory rehabilitation programs, should know their benefits and downsides. They need evidence from RCTs directly comparing respiratory rehabilitation with or without supplements in order to carefully discuss these benefits and downsides with their patients. Therefore, we conducted a systematic review of pragmatic RCTs comparing the effects respiratory rehabilitation with and without any supplemental intervention to assess their added value in HRQL and exercise capacity improvement.

## Methods

### Identification of studies

We performed electronic database searches in MEDLINE (Ovid version, New York, New York, from inception to May 2004), EMBASE (DataStar version, Cary, North Carolina from inception to December 2003), PEDRO (online version, University of Sydney, Australia, December 2003) and the Cochrane Central Register of Controlled Trials (Oxford, United Kingdom, 2003, Issue 4). We also searched the Science Citation Index database (Web of Science, Thomson ISI, Philadelphia, Pennsylvania) and the "related articles" function of PubMed (National Library of Medicine, Washington, Maryland) by entering all included studies. In addition, we hand searched the bibliographies of all included studies, of reviews on respiratory rehabilitation or physical exercise in patients with COPD that were identified in the literature search, as well as the Proceedings of the International Conferences of the American Thoracic Society and the congress of the European Respiratory Society to identify further relevant studies. We also contacted authors in the field to ask for published or unpublished data.

### Selection criteria

We included RCTs investigating any supplemental intervention added to respiratory rehabilitation that included a standardized physical exercise program. We focused on standardized exercise programs because only these allow reproduction in clinical practice. A standardized exercise protocol was defined as the use of an identical exercise activity for all patients (e.g. treadmill walking or cycle ergometer training) at measurable exercise intensity (e.g. in Watts, metabolic equivalents or kilograms). We included studies if more than 90% of study participants patients had COPD according to the following criteria: (1) a clinical diagnosis of COPD, (2) irreversible airways obstruction and (3) one of the following: (a) best recorded FEV1/FVC ratio of individual patients < 0.7; (b) best recorded FEV1 of individual patients < 70% of predicted value. We considered the following outcome measures: HRQL as measured by generic (e.g. SF-36) or disease-specific (e.g. St. George Respiratory Questionnaire) questionnaires, symptom scales, functional exercise capacity as measured walk tests and results from cardiopulmonary exercise testing. We did not apply any language restrictions.

We excluded studies that compared any exercise program versus usual care (i.e. no exercise) or studies that used unstandardised exercise protocols (e.g. home exercise programs).

### Data extraction and quality assessment

**Figure 1:**
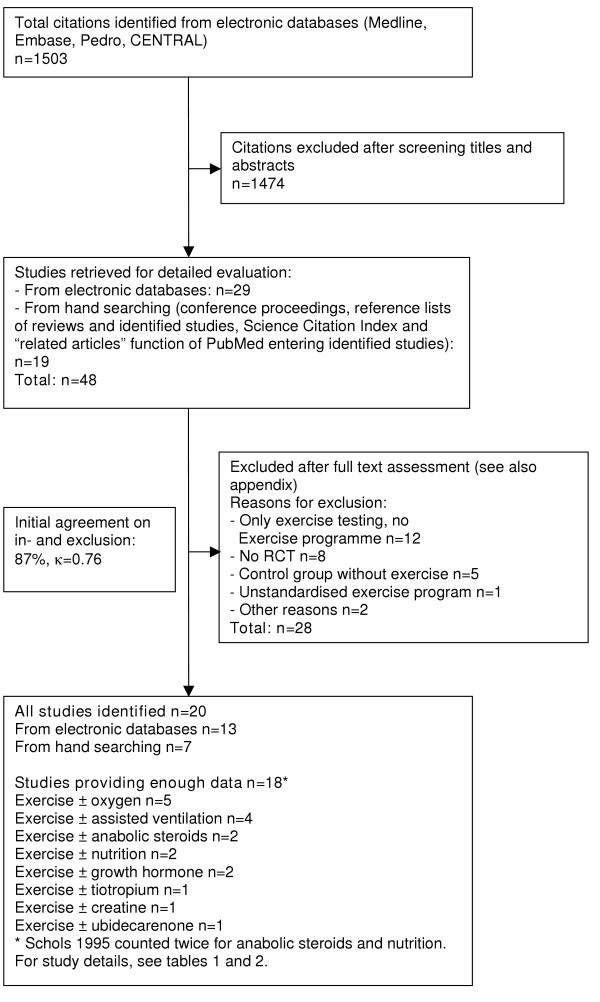
Study flow from identification to final inclusion of studies.

The bibliographic details of all retrieved articles were stored in a Reference Manager file. We removed duplicate records resulting from the various database searches. Two members of the review team independently scrutinized the titles and abstracts of all identified citations (see figure 1). We ordered the full text of any article that was deemed potentially eligible by one of the reviewers. The two reviewers then evaluated the full text of all retrieved papers, made a decision on in- or exclusion and discussed the decisions. Any disagreement was resolved by consensus with close attention to the inclusion and exclusion criteria. Final decisions on papers were recorded in the Reference Manager file and bibliographic details as well as the reasons for exclusion. We recorded the initial degree of agreement between the reviewers and corrected discordant scores based on obvious errors. We resolved discordant scores based on real differences in interpretation through consensus.

Details about study patients, interventions and outcome measures as well as the results were extracted onto a predefined data form. We pilot tested the data forms using five studies with high likelihood for inclusion.

Two reviewers independently evaluated the methodological quality of included trials reported in full reports using a detailed list of quality items assessing components of internal validity[17] (table 3, see Additional file 3). We also contacted the authors of the primary studies to obtain missing information.

### Data synthesis and interpretation

We summarized the results of the data extraction and assessment of study validity in structured tables to allow looking at the variation in patient characteristics, interventions, outcome measures, study quality and results. In addition, we used forest plots to compare results across the trials. If appropriate we planned to explore sources of heterogeneity (i.e. differences between studies) using multivariable regression models (study level meta-regression analysis) where clinical and methodological items would act as explanatory variables. No pooling was undertaken in the presence of significant source heterogeneity.

Whenever possible, for each outcome, estimates and confidence limits was related to its minimal important difference[18]. We assessed whether the estimates and 95% confidence limits for the difference between study groups exceeded the minimal important difference (for the Six-minute walk distance ± 50 meters[19], Chronic Respiratory Questionnaire ± 0.5 points[20] and St. George Respiratory Questionnaire ± 4 points)[21].

Data were analyzed using STATA (version 8.2, Stata Corp., College Station, Texas).

## Results

### Study selection

Figure 1 shows the study selection process and agreement on study inclusion. Main reasons for study exclusion (Appendix, see Additional file 6) were that patients did not have an exercise programme but only exercise testing with or without oxygen (n = 12), studies were not RCTs (n = 8) and that the control group had no exercise programme (n = 5). We excluded only one study because of an undefined exercise programme. We excluded two trials[22,23] from the analysis because the abstract provided little information and the authors did not provide further details. Initially, we excluded another abstract, but since this trial was published in the meantime[24], we could include it in the analysis.

### Quality assessment

Table 3 (see Additional file 3) shows a detailed assessment of the methodological quality of the included trials. Interrater agreement for all items of the quality assessment was 87% (chance corrected agreement: κ = 0.76). In general, most included trials scored poorly on the checklist used. Important methodological aspects that bear on the validity such as blinding of outcome assessors were not or just partially addressed in most trials.

### Supplemental oxygen during exercise

**Figure 2:**
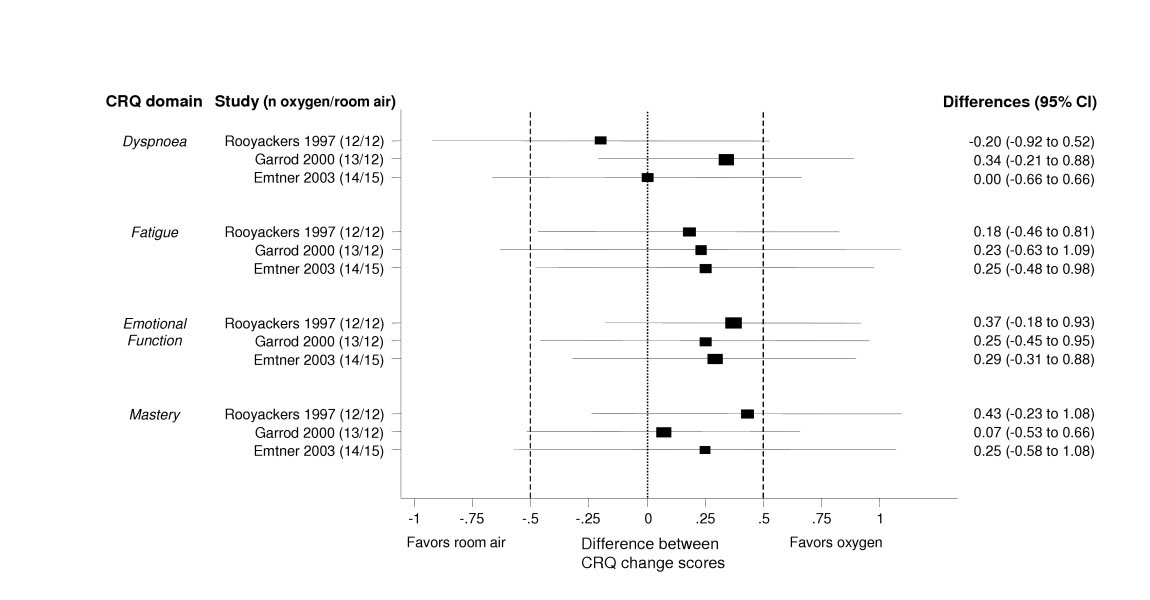
**Effect of supplemental oxygen on health-related quality of life. **The forest plot shows the results from three trials comparing physical exercise with and without oxygen, separately for each domains of the Chronic Respiratory Questionnaire (CRQ). The x-axis represents the difference in change scores between study groups with negative values favoring exercise on room air and positive values favoring exercise with supplemental oxygen. A difference of 0 means that both study groups changed to the same amount. Boxes with 95% confidence intervals represent point estimates for the difference between the CRQ change scores (from baseline to follow-up) of the study groups. Dotted lines represent the minimal important difference of the CRQ (change of 0.5). On the right of the forest plot, point estimates for differences between groups and 95% confidence intervals are shown.

**Figure 3:**
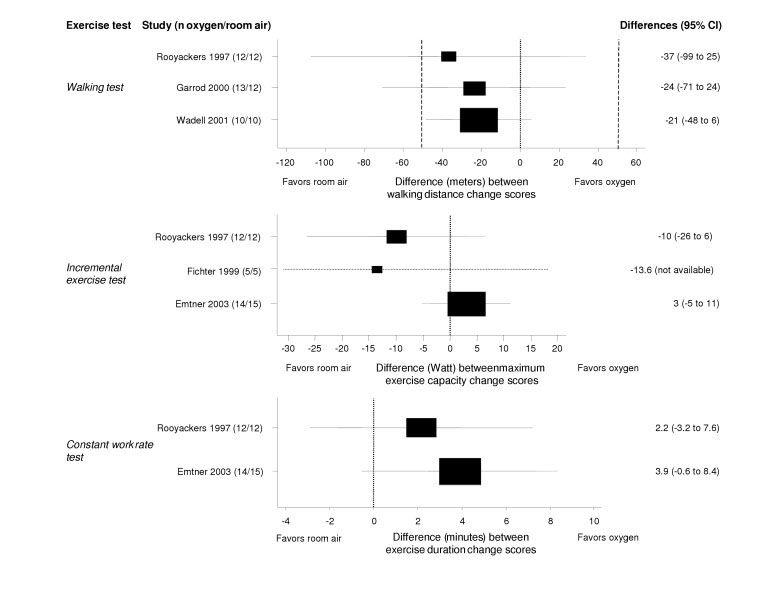
**Effect of supplemental oxygen on exercise capacity. **The forest plot shows the results from five trials comparing respiratory rehabilitation with and without oxygen. Walking tests, incremental and constant work rate exercise tests were used to assess the additional effect of supplemental oxygen during exercise. The x-axis represents the difference in change scores between study groups with negative values favoring exercise on room air and positive values favoring exercise with supplemental oxygen. A difference of 0 means that both study groups changed to the same amount. Boxes with 95% confidence intervals represent point estimates for the difference between the walking distance and maximum exercise capacity change scores (from baseline to follow-up) of the study groups. Dotted lines represent the minimal important difference of the six-minute walking distance (53 meters). On the right of the forest plot, point estimates for differences between groups and 95% confidence intervals are shown.

The characteristics of the five trials on supplemental oxygen [25-29] are summarized in table 1 (see Additional file 1) and the results shown in figures 2 and 3. There was a trend towards larger improvements of HRQL and exercise duration in constant work rate tests in the groups with oxygen, but patients exercising on room air had larger improvements of the walking distance. Emtner[25] reported that the use of oxygen enabled patients to exercise at higher intensity (mean 62 Watt [SD 19] corresponding to 138% of baseline maximum exercise capacity) compared with patients on room air (52 Watt [SD 22] corresponding to 96% of baseline maximum exercise capacity, p < 0.01 for difference between groups). In the trial by Rooyackers[28], patients achieved mean exercise intensities corresponding to 124% of maximum exercise capacity in the group with oxygen and 114% of maximum exercise capacity in the group without oxygen (p = 0.12). Two trials reported on safety of exercise with oxygen or room air. Rooyackers[28] assessed whether oxygen prevented the development of pulmonary hypertension. The investigators did not find any differences between groups in resting mean pulmonary artery pressure measured by Doppler echocardiography. Waddell[29] did not find significant CO_2 _retention during walking tests despite high oxygen flow of 5 l/minute.

### Assisted ventilation

Two trials[30,31] evaluated proportional assist ventilation during exercise and did not find an additional benefit (tables 1 and 4, see Additional files 1 and 4). Only 50%[30] and 71.4%[31] of patients exercising with positive pressure ventilation and 67%[30] and 60%[31] exercising without positive pressure ventilation completed these trials.

Garrod[32] assessed the benefit of overnight non-invasive positive pressure ventilation at home during the training period. They found a statistically significant improvement of the walking distance for patients assigned to overnight non-invasive positive pressure ventilation. HRQL improvements also tended to be larger for patients with ventilation, but the difference reached only statistical significance for the fatigue domain and total score of the CRQ.

Johnson[33] evaluated the effect of ventilation and Heliox during exercise on exercise duration and intensity. They found a small, but statistically not significant increase in exercise duration and intensity for patients exercising with ventilation and Heliox. Patient satisfaction for overall condition, exercise capability and breathing ability measured with global ratings of change did not differ significantly between groups (exact data not available). In this trial, 73.3% of patients with ventilation, 90.9% of patients with Heliox and 84.6% of patients without a supplement completed the trial.

### Nutritional supplements

We identified two RCTs that assess the additional benefit of nutritional supplements during respiratory rehabilitation (table 2, see Additional file 2)[34,35]. Steiner[35] did not find statistically significant differences for HRQL and exercise capacity (table 5, see Additional file 5). In a subgroup of patients with a BMI>19 kg/m^2 ^(22 in group with supplement and 30 in group with placebo) the difference between groups was 27 meters (95% CI 1–53) in the incremental and 121 seconds (95% CI -44–286) in the endurance shuttle walk test. Patients with the carbohydrate-rich diet increased their body weight compared to the placebo group by 1.23 kg (95% CI 0.42–2.05), which occurred mainly because of an increase of the fat mass (difference between groups 1.46 kg, 95% CI 0.65–2.27). There was a dropout rate of 40% in the group with and of 16% in the group without carbohydrate-rich diet.

Another RCT[34] found not significant differences between patients supplemented with an additional fat-rich diet, but did not report the results in detail and could not provide these data for our review. Compared to placebo, non-depleted patients increased their body weight by 1.5 kg (95% CI 0.4–2.6) when receiving a fat-rich diet and by 1.6 kg (95% CI 0.39–2.81) when receiving a fat-rich diet plus anabolic steroids.

### Anabolic steroids

Creutzberg[36] (table 2, see Additional file 2) found that only patients receiving nandrolone improved their HRQL, whereas patients following the respiratory rehabilitation program without nandrolone did not change. This trend was consistent for all domains of the St. George Respiratory Questionnaire, but only statistically significant for the symptom domain (table 5, see Additional file 5). For the subgroup of patients receiving maintenance treatment with oral glucocorticosteroids, patients with nandrolone improved their maximum exercise capacity significantly more. Isometric leg strength and isokinetic legwork improved in both groups, but did not differ significantly between groups. There was a trend in erythropoetic parameters towards an increase of erythrocyte count, hematocrit and hemoglobin in patients treated with nandrolone compared to those treated with placebo. No changes in blood pressures and any androgenic effects or fluid retention were registered in either group.

Casaburi[24] assessed the additional benefit of testosterone for male COPD patients with low testosterone levels who followed a strength exercise program (table 2, see Additional file 2). The group with testosterone had larger increases in exercise capacity and muscle strength, but none of the differences reached statistical significance (table 5, see Additional file 5). Total lean mass increased and total fat mass decreased more in patients with supplemental testosterone, but differences between groups were not significantly different (mean difference in changes between groups in lean mass 3.09 kg, p > 0.05, and total fat mass -1.28 kg, p > 0.05). Casaburi found, like Creutzberg[36], differences in hemoglobin changes between groups (mean difference in hemoglobin change between the testosterone and placebo group 1.4 g/dL, p < 0.05). They observed neither adverse events nor any differences in most safety measures between groups (prostate specific antigen, liver enzymes, alkaline phosphates, cholesterol and high-density lipoprotein cholesterol) between groups. Serum creatinine levels, however, increased in the testosterone group by 0.12 mg/dL and decreased in the placebo group by 0.05 mg/dL (difference between groups 0.17 mg/dL, p < 0.05).

### Tiotropium, Creatine, Coenzyme Q10, and growth hormone

Casaburi[37] assessed the additional benefit of tiotropium in 47 patients and found a significantly increased exercise endurance time compared to patients who received placebo (n = 44, tables 2 and 5, see Additional files 2 and 5). Further results were not available. Four small RCTs evaluated the additional benefit of creatine[38], coenzyme Q10[39] and growth hormone[40,41] during respiratory rehabilitation, but did not find any additional benefit on respiratory or peripheral muscle function or HRQL (tables 2 and 5, see Additional files 2 and 5). Casaburi et al[41] reported that no adverse effects of growth hormone occurred.

## Discussion

There are three main results from this systematic review. First, evidence suggests that supplemental oxygen during physical exercise does not provide a clinically relevant benefit. Second, the evidence for any other supplemental intervention is not strong enough to recommend or discourage their use in clinical practice and third, there were major methodological limitations in most trials that may explain some of the inconclusive findings. We discuss each of these results in turn.

Cotes[42] reported in 1956 that oxygen increased exercise performance in patients with COPD. Since then, many investigators assessed the short-term effect of increased oxygen availability during exercise[15]. Some investigators argue that patients tolerate higher exercise intensities or longer exercise time with supplemental oxygen leading to larger training effects[43,44]. Others believe that only without oxygen, an adequate hypoxemic stimulus is provided for peripheral muscles to improve exercise capacity.

The studies by Emtner[25] and Rooyackers[28] demonstrated that patients indeed tolerate higher exercise intensities if supplemented by oxygen. Mean differences on the CRQ domain scores, however, showed a slight but clinically not meaningful trend towards a benefit with oxygen supplementation (figure 2). The trial by Emtner[25] was the only one that showed a consistent trend towards a small benefit of oxygen on HRQL and exercise capacity. Across all studies, however, results from exercise testing were contradicting. Supplemental oxygen did prolong exercise duration in constant work rate tests, but led to considerably smaller improvements of functional exercise capacity (figure 3). It was hypothesized earlier that those patients with the highest oxygen desaturation during exercise would benefit most from supplemental oxygen[45]. The trials do not provide sufficient evidence for or against this hypothesis.

There is limited evidence on the safety of oxygen during exercise and on the safety of exercise without oxygen in patients with desaturation. Clinicians may have concerns about training in hypoxemia because of adverse events and will encourage oxygen supplementation in patients with desaturation during exercise. In theory, oxygen carries the risk of CO_2 _retention in COPD patients. The only trial reporting on CO_2 _retention[29] did not observe significant differences of CO_2 _levels during exercise tests with oxygen compared with exercise on room air. However, exercise tests may have been too short to assess the effect of CO_2 _retention. Exercise is a risk indicator for unmasking latent pulmonary hypertension[46], but supplemental oxygen may reduce this risk by decreasing the sympathetic tone and the respiratory rate allowing for less end-expiratory pressure[47]. Rooyackers[28] did not find any differences in resting mean pulmonary artery pressure between patients with and without oxygen. However, patients stopped exercising when oxygen saturation fell below 90% so that the risk of the exercise program under hypoxemic conditions on the development of pulmonary hypertensions could not be studied.

Several studies found a positive acute effect of oxygen during exercise testing on exercise capacity and a number of physiologic mechanisms for the effects of oxygen have been proposed [48-50]. However, these results on the short-term benefit of oxygen during exercise testing seem not to translate into improvements of clinically relevant outcomes during exercise programs. Current data do not suggest benefit from the use of oxygen during exercise to enhance training effects (figure 3), but show some benefit in terms of HRQL (figure 2) Given the limited methodological quality of trials, any conclusions are vague. The general use of oxygen is only justified, if larger trials of good quality show its benefit on clinically relevant outcomes. The mechanisms of the effects of oxygen during exercise are still insufficiently understood and call for more basic research[15].

Assisted ventilation also aims at increasing oxygen availability during exercise, but the trials indicated no additional benefit. An exception may represent overnight non-invasive positive pressure ventilation. This treatment may improve quality of sleep as well as daytime gas levels and respiratory muscle function thereby providing a better milieu (pH, PaO_2_, PaCO_2_) for peripheral muscle function. One trial[32] found statistically significant improvements of functional exercise capacity and also large improvements of HRQL (mean differences between groups 0.45 to 0.85 in CRQ domain scores, table 4, see Additional file 4) with additional non-invasive positive pressure ventilation. These results support the hypothesis formulated by authors of a recent meta-analysis showing that nocturnal non-invasive positive pressure ventilation alone has no effect on exercise capacity and HRQL, but may be beneficial as an adjunct to respiratory rehabilitation[51]. The eight trials that assessed various supplemental interventions during rehabilitation produced inconclusive results that do not allow recommendations for clinical practice yet.

An important result of this systematic review with implications for future research is the low methodological quality and small sample sizes. For example, the majority of trials did not consider stratification for important prognostic factors such as exercise capacity[52] for randomization. In some trials there were baseline imbalances between groups, for example in terms of exercise capacity[27,28,32,33,40]. The influence of these imbalances on the results was not investigated in any of the trials. Concealment of random allocation and blinding of treatment providers or outcome assessors was also not addressed in most trials.

**Figure 4:**
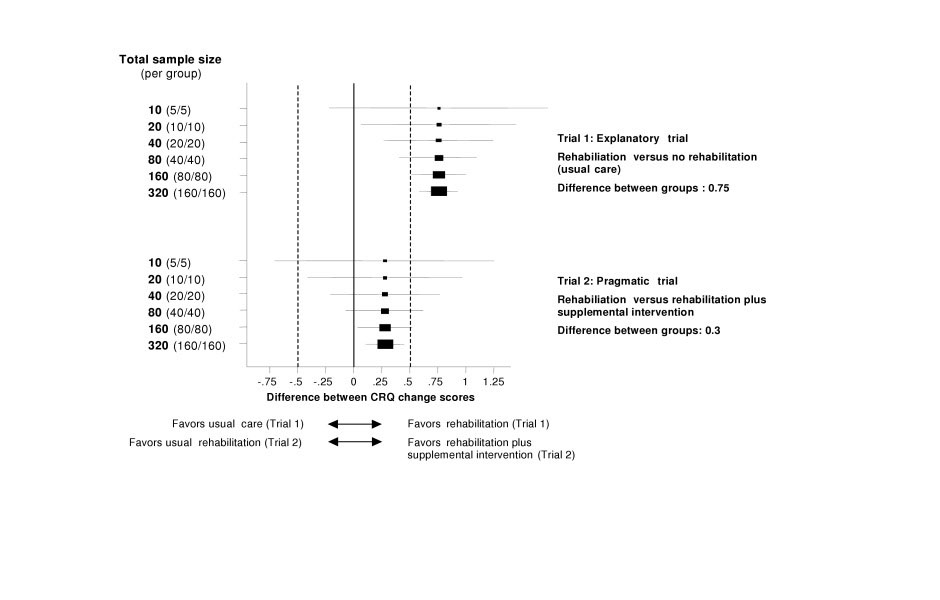
**Sample size and interpretation of randomized controlled trials in respiratory rehabilitation. **Forest plot with simulated results from two trials with varying sample size, in which the CRQ was used. Boxes with 95% confidence intervals represent point estimates for the difference between CRQ change scores (from baseline to follow-up) of the study groups. Dotted lines represent the minimal important difference of the CRQ (change of 0.5). Trial 1 shows the results from a typical explanatory trial comparing respiratory rehabilitation and no respiratory rehabilitation (usual care) with differences in CRQ change scores around 0.75[5]. Because of the large effect, trial results are interpretable also with imprecise results. Trial 2 shows the results from a pragmatic trial assessing the additional effect of a supplemental intervention (for example oxygen during exercise). The difference between groups is 0.3 and sample size must be large (80 per group) to produce results that are precise enough to allow interpretation.

Sample sizes were small except in three trials[34,35,37]. Pragmatic trials comparing active interventions, as included in this systematic review, are very useful for clinical practice when clinicians are confronted with the choice between interventions[53]. However, small sample sizes are problematic in pragmatic trials for at least two reasons: First, differences between study groups tend to be smaller in pragmatic trials than in trials comparing an active intervention with placebo or a sham intervention. Figure 4 shows the results and 95% confidence intervals of a trial comparing respiratory rehabilitation with usual care and of a trial comparing respiratory rehabilitation with and without a supplemental intervention with different sample sizes. It illustrates the importance of sufficient sample sizes in pragmatic trials by showing that for pragmatic RCTs in respiratory rehabilitation, in which widely established patient-important outcomes such as HRQL are used, sample sizes of up to 40 per group will produce imprecise results (large confidence intervals). This imprecision hinders interpretation. Another reason for sufficient samples sizes is that in pragmatic trials patient profiles are usually more variable than in explanatory trials reflecting the wide patient spectrum encountered in clinical practice[53]. The greater variability in patient profiles carries, on one side, a greater risk for confounding and, on the other side, subgroup analyses will be important to assess whether the effects differ between patient subgroups (effect modification). Subgroup analyses based on prognostically important patient characteristics will provide more differentiated evaluations than one mean for the whole study group, but they require sufficient sample for well-balanced intervention groups.

We propose that investigators consider the following aspects in future pragmatic trials on respiratory rehabilitation: First, preliminary sample size considerations should be based on realistic estimates for expected differences between groups, which are typically smaller than in trials without active comparators. To better understand what these sample sizes mean, 95% confidence intervals around the predicted point estimate can be calculated as shown in figure 4. This approach will help to better foresee the consequence of different sample sizes on interpretation of the data[54]. Second, COPD patients represent a heterogeneous group and stratification for prognostically important variables should be considered to avoid baseline imbalances that bear on outcomes[55], as seen in some trials included in this review[27,28,32,33,40]. Third, more attention needs to be paid to general requirements for RCTs of high quality like method of randomisation, concealment of random allocation and blinding of those who assess treatment effects.

The strengths of our systematic review include the broad literature search including several databases and extensive hand searching for trials with direct comparisons of interventions that are used in clinical practice. In addition, we contacted authors for additional data and received them from the majority of investigators. This greatly enhanced the informativeness of included studies and thereby of this review. A weakness of this review includes the discussion that is limited to the best-investigated supplements because of the number of interventions included in this review. However, the aim of this review was to analyze current evidence from a meta-epidemiological perspective not giving to much emphasis to single studies. Some may criticize that we did not pool the results from trials on supplemental oxygen during exercise using meta-analysis. However, desaturation or no desaturation during exercise was an important inclusion criterion in four of the five trials and investigators wanted to learn about the effect of supplemental oxygen in these subgroups, in particular. Therefore, we considered the patient profiles of these trials to be too different to provide meaningful pooled estimates. Instead, we provided forest plots (figures 2 and 3) to show the individual studies' point estimates and 95% confidence intervals for the CRQ domains and the exercise tests to allow comparisons across studies.

In conclusion, data for most supplemental interventions during respiratory rehabilitation are inconclusive. Oxygen during exercise does not seem to provide a patient-important additional benefit for COPD patients during a respiratory rehabilitation, but methodological shortcomings of the trials on supplemental oxygen do not allow conclusive answers. Future trials should pay careful attention to the methodological trial design and to sufficient sample sizes.

## Abbreviations

COPD: Chronic obstructive pulmonary disease

RCT: Randomised controlled trial

HRQL: Health-related quality of life

CRQ: Chronic Respiratory Questionnaire

## Conflict of interest

The authors declare that they have no competing interests.

## Contributions

Protocol writing: Puhan, Bachmann, Schunemann

Acquisition of data: Puhan, Bachmann

Analysis and interpretation of data: Puhan, Bachmann, Schunemann, Frey

Drafting of manuscript: Puhan, Bachmann

Critical revision of manuscript for important intellectual content: Puhan, Bachmann, Schunemann, Frey

## Funding

Helmut Horten Foundation

Lucas M. Bachmann: Swiss National Science Foundation Research Fellow (PROSPER programme)

## Supplementary Material

Additional File 3 Table 3: Internal validity of included studies Click here for file

Additional File 6 Appendix: The appendix lists all studies that were excluded after full text assessment. The full reference and the reason for exclusion are given. Click here for file

Additional File 1 Table 1: Characteristics of randomised controlled trials investigating supplemental oxygen and assisted ventilation Click here for file

Additional File 4 Table 4: Effect of assisted ventilation on HRQL and exercise capacity Click here for file

Additional File 2 Table 2: Characteristics of randomised controlled trials investigating drug and nutritional supplements Click here for file

Additional File 5 Table 5: Effect of drug and nutritional interventions on HRQL and exercise capacity Click here for file
