# Supplementary material for: Value of supplemental interventions to enhance the effectiveness of physical exercise during respiratory rehabilitation in COPD patients. A Systematic Review
Source: Respir Res. 2004 Dec 2;5(1):25. doi: 10.1186/1465-9921-5-25 (PMC539299; doi:10.1186/1465-9921-5-25)
Supplement: Additional File 3 — Table 3: Internal validity of included studies [file 1465-9921-5-25-S3.doc]

Table 3: Internal validity of included studies

| Study | Prognostically homogenous study population | Con-cealment of random allocation | Prestratification on prognostically relevant variables | Description of randomisation procedure | Registration of loss to follow-up | Blinding of patients | Blinding of persons who implement interventions | Registration of co-interventions that bear on outcome for each group | Blinding of persons who assess treatment effects | Check to what extent blinding was successful |
| --- | --- | --- | --- | --- | --- | --- | --- | --- | --- | --- |
| Bianchi 2002[30] | + | - | - | - | + | - | - | - | - | - |
| Burdet 1997[40] | +/- | - | - | +/- | - | +/- | +/- | - | - | - |
| Casaburi 2004[24] | + | - | + | +/- | + | + | + | - | - | - |
| Creutzberg 2003[36] | + | +/- | - | +/- | + | + | + | +/- | +/- | - |
| Emtner 2003[25] | +/- | +/- | - | - | - | +/- | +/- | +/- | - | - |
| Fichter 1999[26] | + | - | - | - | - | - | - | +/- | - | - |
| Garrod 2000[27] | +/- | +/- | - | - | - | +/- | - | - | - | - |
| Garrod 2000[32] | + | +/- | - | - | + | - | - | - | - | - |
| Hawkins 2002[31] | +/- | +/- | - | - | +/- | - | - | +/- | - | - |
| Johnson 2002[33] | +/- | - | - | - | +/- | - | - | +/- | - | - |
| Rooyackers 1997[28] | + | - | - | - | - | - | - | - | - | - |
| Satta 1994[39] | +/- | - | - | - | + | - | - | - | - | - |
| Schols 1995[34] | + | - | + | - | +/- | +/-^2^ | +/-^2^ | +/- | - | - |
| Steiner 2003[35] | + | + | - | +/- | + | + | + | + | +/- | - |
| Wadell 2001[29] | + | - | +/- | - | +/- | +/- | - | +/- | - | - |

+ Item properly addressed; +/- Item partially addressed; - Item not addressed

^1^ 15 of 20 trials were included in the quality assessment. 5 trials were only available as abstracts and provided little details on internal validity.

^2^ Applies only to nandrolone and placebo for nandrolone. For nutritional supplement, neither patients nor persons who implement intervention were blinded.
