# Supplementary material for: Value of supplemental interventions to enhance the effectiveness of physical exercise during respiratory rehabilitation in COPD patients. A Systematic Review
Source: Respir Res. 2004 Dec 2;5(1):25. doi: 10.1186/1465-9921-5-25 (PMC539299; doi:10.1186/1465-9921-5-25)
Supplement: Additional File 6 — Appendix: The appendix lists all studies that were excluded after full text assessment. The full reference and the reason for exclusion are given. [file 1465-9921-5-25-S6.doc]

Appendix: Excluded studies after full text assessment

| **Study** | **Reason for exclusion** |
| --- | --- |
| Bianchi 1998(1) | Not randomised controlled trial |
| BourjeilyHabr 2002(2) | Not randomised controlled trial |
| Bradley 1978(3) | Control group without exercise |
| Costes 2003(4) | Not randomised controlled trial |
| Creutzberg 2003(5) | Not randomised controlled trial |
| Dolmage 1997(6) | No exercise program |
| Ferreira 1998(7) | No exercise throughout whole study |
| Goris 2003(8) | Control group without exercise |
| Gort 1993(9) | Control group without exercise |
| Gosselink 1997(10) | Not randomised controlled trial |
| Hernandez 2001(11) | No exercise program |
| Jolly 2001(12) | No exercise program |
| Koechlin 2004(13) | Only localized quadriceps exercise, no rehabilitative exercise program |
| Kurihara 1989(14) | No exercise programme |
| Maa 1997(15) | Undefined exercise program |
| McDonald 1995(16) | No exercise program |
| McKeon 1988(17) | No exercise program |
| Nandi 2003(18) | No exercise program |
| Palange 1993(19) | Not randomised controlled trial |
| Polkey 2000(20) | No exercise program |
| Pollini 1981(21) | Not randomised controlled trial |
| Revill 2000(22) | No exercise program |
| Rogers 1992(23) | Control group without exercise |
| Slinde 2002(24) | Not randomised controlled trial |
| Teramoto 1995(25) | No exercise program |
| Teramoto 1996(26) | No exercise program |
| Weiner 2000(27) | Control group without exercise |
| Young 1989(28) | No exercise program |

Reference List Appendix

1. Bianchi L, Foglio K, Pagani M, Vitacca M, Rossi A, Ambrosino N. Effects of proportional assist ventilation on exercise tolerance in COPD patients with chronic hypercapnia. European Respiratory Journal 1998;11(2):422-7.
2. BourjeilyHabr G, Rochester CL, Palermo F, Snyder P, Mohsenin V. Randomised controlled trial of transcutaneous electrical muscle stimulation of the lower extremities in patients with chronic obstructive pulmonary disease. Thorax 2002;57(12):1045-9.
3. Bradley BL, Garner AE, Billiu D, Mestas JM, Forman J. Oxygen-assisted exercise in chronic obstructive lung disease. The effect on exercise capacity and arterial blood gas tensions. Am Rev.Respir Dis. 1978;118(2):239-43.
4. Costes F, Agresti A, CourtFortune I, Roche F, Vergnon JM, Barthelemy JC. Noninvasive ventilation during exercise training improves exercise tolerance in patients with chronic obstructive pulmonary disease. Journal of Cardiopulmonary Rehabilitation 2003;23(4):307-13.
5. Creutzberg EC, Wouters EFM, Mostert R, WelingScheepers CAPM, Schols AMWJ. Efficacy of nutritional supplementation therapy in depleted patients with chronic obstructive pulmonary disease. Nutrition 2003;19(2):120-7.
6. Dolmage TE, Goldstein RS. Proportional assist ventilation and exercise tolerance in subjects with COPD. Chest 1997;111(4):948-54.
7. Ferreira IM, Verreschi IT, Nery LE, Goldstein RS, Zamel N, Brooks D et al. The influence of 6 months of oral anabolic steroids on body mass and respiratory muscles in undernourished COPD patients. Chest 1998;114(1):19-28.
8. Goris AH, Vermeeren MA, Wouters EF, Schols AM, Westerterp KR. Energy balance in depleted ambulatory patients with chronic obstructive pulmonary disease: the effect of physical activity and oral nutritional supplementation. British Journal of Nutrition 2003;89(5):725-31.
9. Gort EH, Goldstein R, Guyatt G, Stubbing D, Avendano M. Randomized controlled trial of respiratory rehabilitation. Canadian Journal of Rehabilitation 1993;7(1):13-4.
10. Gosselink R, Troosters T, Decramer M. Exercise training in COPD patients: the basic questions. Eur.Respir J 1997;10(12):2884-91.
11. Hernandez P, Maltais F, Gursahaney A, LeBlanc P, Gottfried SB. Proportional assist ventilation may improve exercise performance in severe chronic obstructive pulmonary disease. Journal of Cardiopulmonary Rehabilitation 2001;21(3):135-42.
12. Jolly EC, Di B, V, Aguirre L, Luna CM, Berensztein S, Gene RJ. Effects of supplemental oxygen during activity in patients with advanced COPD without severe resting hypoxemia. Chest 2001;120(2):437-43.
13. Koechlin C, Couillard A, Simar D, Cristol JP, Bellet H, Hayot M et al. Does oxidative stress alter quadriceps endurance in chronic obstructive pulmonary disease? Am J Respir Crit Care Med 2004;169(9):1022-7.
14. Kurihara N, Fujimoto S, Kohno M, Ohta K, Hirata K, Takeda T. Exercise induced hypoxemia and exercise tolerance in patients with COPD and the benefits of oxygen supplementation. [Japanese]. Nihon Kyobu Shikkan Gakkai Zasshi 1989;27(2):155-62.
15. Maa SH, Gauthier D, Turner M. Acupressure as an adjunct to a pulmonary rehabilitation program. Journal of Cardiopulmonary Rehabilitation 1997;17(4):268-76.
16. McDonald CF, Blyth CM, Lazarus MD, Marschner I, Barter CE. Exertional oxygen of limited benefit in patients with chronic obstructive pulmonary disease and mild hypoxemia. American Journal of Respiratory & Critical Care Medicine 1995;152(5:Pt 1):t-9.
17. McKeon JL, MurreeAllen K, Saunders NA. Effects of breathing supplemental oxygen before progressive exercise in patients with chronic obstructive lung disease. Thorax 1988;43(1):53-6.
18. Nandi K, Smith AA, Crawford A, MacRae KD, Garrod R, Seed WA et al. Oxygen supplementation before or after submaximal exercise in patients with chronic obstructive pulmonary disease. Thorax 2003;58(8):670-3.
19. Palange P, Forte S, Felli A, Carlone S. Nutritional status and exercise performance in chronic obstructive pulmonary disease (COPD). Monaldi Archives for Chest Disease 1993;48(5):543-5.
20. Polkey MI, Hawkins P, Kyroussis D, Ellum SG, Sherwood R, Moxham J. Inspiratory pressure support prolongs exercise induced lactataemia in severe COPD. Thorax 2000;55(7):547-9.
21. Pollini G, Biscaldi G, Prestinoni A. Value of salbutamol aerosol administration by intermittent positive pressure in rehabilitation of patients with chronic obstructive bronchopneumopathies. [Italian]. Giornale Italiano di Medicina del Lavoro 1981;3(1):41-4.
22. Revill SM, Singh SJ, Morgan MD. Randomized controlled trial of ambulatory oxygen and an ambulatory ventilator on endurance exercise in COPD. Respiratory Medicine 2000;94(8):778-83.
23. Rogers RM, Donahoe M, Costantino J. Physiologic effects of oral supplemental feeding in malnourished patients with chronic obstructive pulmonary disease. A randomized control study. American Review of Respiratory Disease 1992;146(6):1511-7.
24. Slinde F, Gronberg AM, Engstrom CR, RossanderHulthen L, Larsson S. Individual dietary intervention in patients with COPD during multidisciplinary rehabilitation. Respiratory Medicine 2002;96(5):330-6.
25. Teramoto S, Fukuchi Y. Improvements in exercise capacity and dyspnoea by inhaled anticholinergic drug in elderly patients with chronic obstructive pulmonary disease. Age & Ageing 1995;24(4):278-82.
26. Teramoto S, Matsuse T, Sudo E, Ohga E, Katayama H, Suzuki M et al. Long-term effects of inhaled anticholinergic drug on lung function, dyspnea, and exercise capacity in patients with chronic obstructive pulmonary disease. Internal Medicine 1996;35(10):772-8.
27. Weiner P, Magadle R, BerarYanay N, Davidovich A, Weiner M. The cumulative effect of long-acting bronchodilators, exercise, and inspiratory muscle training on the perception of dyspnea in patients with advanced COPD. Chest 2000;118(3):672-8.
28. Young IH, Daviskas E, Keena VA. Effect of low dose nebulised morphine on exercise endurance in patients with chronic lung disease. Thorax 1989;44(5):387-90.
