# Supplementary material for: Value of supplemental interventions to enhance the effectiveness of physical exercise during respiratory rehabilitation in COPD patients. A Systematic Review
Source: Respir Res. 2004 Dec 2;5(1):25. doi: 10.1186/1465-9921-5-25 (PMC539299; doi:10.1186/1465-9921-5-25)
Supplement: Additional File 1 — Table 1: Characteristics of randomised controlled trials investigating supplemental oxygen and assisted ventilation [file 1465-9921-5-25-S1.doc]

Table 1: Characteristics of randomised controlled trials investigating supplemental oxygen and assisted ventilation

|  | **Total sample size** | **Mean age (years)** | **Desaturation during exercise and PaO_2_ at rest** | **Mean FEV1 % predict.** | **Exercise program** | **Exercise session (min) and times/week** | **Duration exercise program** | **Supplemental intervention** | **Additional interventions in both groups** | **Outcomes** |
| --- | --- | --- | --- | --- | --- | --- | --- | --- | --- | --- |
| Emtner 2003[25] | 29  (62% males) | 67 | Only patients without desaturation  (Sp_02_ ≥ 88%)  PaO_2_ = 9.7 kPa | 36.6 | ET: Continuous high intensity cycling at 80% of Wmax for 35 min (+ 5 min warming up and cooling down) | 45 min  3x/week | 7 weeks | 3 L oxygen per minute | Edu | IET, CWRT, CRQ, SF-36 |
| Fichter 1999[26] | 10  (100% males) | 59 | Patients ± desaturation  PaO_2_ = 9.9 kPa | 43.2 | ET: Continuous high intensity cycling at 80% of Wmax for 45 min | 45 min  5x/week | 4 weeks | 3.5 L oxygen per minute | No | IET |
| Garrod 2000[27] | 25  (72% males) | 67 | Patients with desaturation during exercise (Sp_02_ fall of 4% or ≤ 90%)  PaO_2_ = 8.5 kPa | 31.6 | ET: Continuous high intensity walking at 80% of maximum oxygen consumption in SWT and low intensity cycling (unloaded)  ST: Upper and lower extremity | 60 min  3x/week | 6 weeks | 4 L oxygen per minute | Edu, BE, Psy, Re | SWT, CRQ, HADS |
| Rooyackers 1997[28] | 24  (83% males) | 61 | Patients with desaturation during exercise  (Sp_02_ ≤ 90%)  PaO_2_ = 10.4 kPa | 33.5 | ET: Interval cycle exercise (2 min exercise /2 min rest)  ST: Upper and lower extremity | 50 min  5x/week | 10 weeks | 4 L oxygen per minute. Patients stopped exercising when Sp_02_ ≤ 90% | Edu, Psy, Re | IET, CWRT, 6MWT, CRQ |
| Wadell 2001[29] | 20  (50% males) | 67 | Only patients with desaturation  (Sp_02_ ≤ 92%)  PaO_2_ = 9.4 kPa | 45.3 | ET: Interval treadmill exercise (2 min high speed with target dyspnea of 7/10 on Borg/2 min low speed) | 30 min  3x/week | 8 weeks | 5 L oxygen per minute | No | 6MWT |
| Bianchi 2002[30] | 33  (100% males) | 64.5 | Patients ± desaturation  PaO_2_ = 10.0 | 44.2 | ET: Moderate intensity cycling at 50-70% of Wmax  ST: Upper and lower extremity | 30 min  3x/week | 6 weeks | Proportional assist ventilation during exercise | Edu | 6MWT, IET, SGRQ, BDI, TDI |
| Garrod 2000[32] | 45  (62% males) | 65 | Patients ± desaturation  PaO_2_ = 8.7 | 34.1 | ET: Walking at 80% of VO_2_max of SWT + low intensity cycling  ST: Upper and lower extremity | 60 min  2x/week | 8 weeks | Overnight non-invasive positive pressure ventilation during training period | Edu, Rel | SWT, CRQ, HADS, LCADL |
| Hawkins 2002[31] | 19  (89% males) | 67 | Patients ± desaturation  PaO_2_ = 8.4 | 26.9 | ET: High intensity cycling at 70% of Wmax | 30 min  3x/week | 6 weeks | Proportional assist ventilation during exercise | No | IET |
| Johnson 2002[33] | 32  (68 % males) | 69 | Patients ± desaturation  PaO_2_ = 9.4 | 32.1 | ET: high intensity treadmill walking at 50-60% of METmax at baseline | 20 min  2x/week | 6 weeks | Noninvasive positive pressure ventilation during exercise  Heliox 10l/min | Edu, BE, Psy, Rel | IET, exercise duration, global ratings |

ET: Endurance training; ST: Strength training; Wmax: maximum exercise capacity; SWT: Incremental shuttle walk test; MET: Metabolic equivalent; Edu: education; BE: breathing exercises; Psy: psychological support; Rel: Relaxation exercises; IET: Incremental exercise test; CWRT: Constant work rate test; CRQ: Chronic Respiratory Questionnaire; SF-36: Short from survey; HADS: Hospital Anxiety Depression Scale; 6MWT: 6-Minute walk test; SGRQ: St George Respiratory Questionnaire; ESWT = Endurance shuttle walk test; BDI and TDI = Baseline and transitional dyspnea index; LCADL: London Chest Activity of Daily Living Scale
