# Supplementary material for: Value of supplemental interventions to enhance the effectiveness of physical exercise during respiratory rehabilitation in COPD patients. A Systematic Review
Source: Respir Res. 2004 Dec 2;5(1):25. doi: 10.1186/1465-9921-5-25 (PMC539299; doi:10.1186/1465-9921-5-25)
Supplement: Additional File 4 — Table 4: Effect of assisted ventilation on HRQL and exercise capacity [file 1465-9921-5-25-S4.doc]

Table 4: Effect of assisted ventilation on HRQL and exercise capacity

| Study | **Outcome measure** | **Difference between intervention and control group (95% CI where available)** |
| --- | --- | --- |
| Bianchi 2002[30] (n_1_=9;n_2_=10) | SGRQ Total | 2.8 (-2.5 to 8.1) |
|  | SGRQ Symptom | 3.4 (-11.8 to 18.7) |
|  | SGRQ Activity | 7.3 (-1.5 to 16.1) |
|  | SGRQ Impact | 0.1 (-7.8 to 8.1) |
|  | Transitional dyspnea index | 0.1 (-2.4 to 2.6) |
|  | Functional exercise capacity (walking distance in meters) | -31 (-68 to 24) |
|  | Maximum exercise capacity (in Watt) | 6.0 (-12.3 to 24.3) |
| Garrod 2000[32]  (n_1_=17;n_2_=20) | CRQ total | 0.62 (0.06 1.17) |
|  | CRQ Dyspnea | 0.66 (-0.25 to 1.57) |
|  | CRQ Fatigue | 0.85 (0.2 to 1.52) |
|  | CRQ Emotional function | 0.54 (-0.53 to 0.92) |
|  | CRQ Mastery | 0.45 (-0.32 to 1.21) |
|  | HADS total | -2.51 (4.98 to –9.58) |
|  | HADS anxiety | -0.66 |
|  | HADS depression | -1.82 |
|  | LCADL total | 0.3 (-5.69 to 6.4) |
|  | LCADL self care | 0.4 |
|  | LCADL domestic | -1.9 |
|  | LCADL physical | 0.65 |
|  | LCADL leisure | 1.1 |
|  | Functional exercise capacity (walking distance in meters) | 72 (13 to 131) |
| Hawkins 2002[31]  (n_1_=10;n_2_=9) | Maximum exercise capacity (in Watt) | 7.1 (2.9 to 11.3) |
|  | Maximum exercise duration (in minutes) | 2.4 |
| Johnson 2002[33]  (n_1_=11;n_2_=11;  n_3_=10) | Maximum work load (in METs)  NIPPV versus control (room air)  Heliox versus control (room air) | 0.18  0.16 |
|  | Treadmill walking exercise duration (in minutes)  NIPPV versus room air  Heliox versus room air | 2.6  2.3 |

n_1_= Intervention group;n_2_= Control group, n_3_= Heliox group

MET: Metabolic equivalents, NIPPV: Non-invasive positive pressure ventilation

CRQ, maximum and functional exercise capacity: Between group differences > 0 favors intervention group; HADS, SGRQ, LCADL Breathlessness at maximum exercise capacity (Borg scale): Between group differences > 0 favors control group
