# Supplementary material for: Value of supplemental interventions to enhance the effectiveness of physical exercise during respiratory rehabilitation in COPD patients. A Systematic Review
Source: Respir Res. 2004 Dec 2;5(1):25. doi: 10.1186/1465-9921-5-25 (PMC539299; doi:10.1186/1465-9921-5-25)
Supplement: Additional File 2 — Table 2: Characteristics of randomised controlled trials investigating drug and nutritional supplements [file 1465-9921-5-25-S2.doc]

Table 2: Characteristics of randomised controlled trials investigating drug and nutritional supplements

|  | **Total sample size** | **Mean age (years)** | **Mean FEV1 % predict.** | **Exercise program** | **Exercise session (min) and times/week** | **Duration exercise program** | **Supplemental intervention** | **Additional interventions in both groups** | **Outcomes** |
| --- | --- | --- | --- | --- | --- | --- | --- | --- | --- |
| Burdet 1997[40] | 16  (87.5% males) | 66 | 39.5 | ET and ST (no details available) | 45 min  6-7x/week | 3 weeks | Daily injections of 0.15 IU/kg recombinant human growth hormone s.c. vs. placebo | Edu, Psy | IET, 6MWT, HADS, body composition |
| Casaburi 1997[41] | 29  (31% males) | 69 | 42.0 | ET: Continuous high intensity cycling at 85% of Wmax | 45 min  3x/week | 6 weeks | 40 μg/kg growth hormone s.c. vs placebo | Unclear | IET, CWRT, body composition, respiratory muscle strength and endurance, quality of life (instrument unclear) |
| Casaburi 2004[37] | 91  (57% males) | 67 | 34.0 | ET: Cycling (no details available) | ≥30 min  3x/week | 8 weeks | 18 μg tiotropium/day 5 weeks prior, during and 12 weeks following rehabilitation | Unclear | Endurance time during treadmill walking at 80% of initial IET |
| Casaburi 2004[24] | 23 (100% males) | 67 | 39.0 | ST: 4 leg exercises with 10-12 repetitions at 60-80% of one repetition maximum | 45 min  3x/week | 10 weeks | 100 mg testosterone enanthate i.m. per week. Target testosterone level: 450-850 ng/dl. If level <400 ng/dl increase to 125 mg per week and if above 800 ng/dl decrease to 75 mg per week | Diet with 55% carbohydrates, 30% fat, 15% protein and 100% of daily requirements for vitamins and minerals | IET, CWRT, quadriceps force, quadriceps fatigability, body composition, erythropoetic and endocrine parameters |
| Creutzberg 2003[36] | 56  (100 % males) | 67.5 | 35.5 | ET: Cycling (intensity determined by IET but unclear), treadmill walking, swimming | 40 min  5x/week | 8 weeks | 50mg nandrolone decanoate i.m. at day 1, 15, 29 and 43 vs. placebo | Edu, nutrition supplements for depleted patients | IET, SGRQ, handgrip force, body composition, erythropoetic and endocrine parameters |
| Gosselink 2003[38] | 26 | 65 | 42.0 | ET and ST | Unclear  3/week | 12 weeks | 4x5g creatine in first week, followed by 5g/day for 11 weeks vs. placebo | Unclear | 6MWT, IET, quadriceps force, |
| Satta 1994[39] | 20  (80% males) | 69 | 50.1 | ET: Treadmill walking (intensity determined by IET but unclear) | 30 min  5x/week | 4-6 weeks | 50 mg Coenzyme Q10 (ubidecarenone) per os per day vs. placebo | Edu, BE, Rel | IET |
| Schols 1995[34] | 217  (% males unclear) | No details available | 33.8 | ET: Cycling, treadmill walking, circuit walking, swimming | 40 min  5x/week | 8 weeks | 1. Additional 402 kcal per day with 35% carbohydrates, 51% fat and 14% proteins.  2. 50mg (men) or 25 mg (women) nandrolone decanoate i.m. at day 1, 15, 29 and 43 | Optimization of drug therapy  Unclear if Edu, Psy, Rel or BE | 12MWT, body composition |
| Steiner 2003[35] | 85  (62% males) | 67 | 34.5 | ET: High intensity walking at 85% of VO_2_max of SWT. Low impact conditioning exercises. | Duration unclear  2x/week | 7 weeks | Additional 570 kcal per day with 60% carbohydrates, 20% fat and 20% proteins vs. placebo non-nutritive placebo diet | Edu, Psy, home walking training | SWT, ESWT, CRQ, muscle force, body composition |

ET: Endurance training; ST: Strength training; Wmax: maximum exercise capacity; SWT: Incremental shuttle walk test; Edu: education; BE: breathing exercises; Psy: psychological support; Rel: Relaxation exercises; IET: Incremental exercise test; CWRT: Constant work rate test, CRQ: Chronic Respiratory Questionnaire; SF-36: Short from survey; HADS: Hospital Anxiety Depression Scale; 6MWT: 6-Minute walk test; SGRQ: St George Respiratory Questionnaire; ESWT = Endurance shuttle walk test; BDI and TDI = Baseline and transitional dyspnea index; LCADL: London Chest Activity of Daily Living Scale
