# Supplementary material for: Value of supplemental interventions to enhance the effectiveness of physical exercise during respiratory rehabilitation in COPD patients. A Systematic Review
Source: Respir Res. 2004 Dec 2;5(1):25. doi: 10.1186/1465-9921-5-25 (PMC539299; doi:10.1186/1465-9921-5-25)
Supplement: Additional File 5 — Table 5: Effect of drug and nutritional interventions on HRQL and exercise capacity [file 1465-9921-5-25-S5.doc]

Table 5: Effect of drug and nutritional interventions on HRQL and exercise capacity

|  | **Outcome measure** | | **Difference between intervention and control group* (95% CI where available)** |
| --- | --- | --- | --- |
| Burdet 1997[40]  (n_1_=8;n_2_=8) | Functional exercise capacity (walking distance in meters) | | -102 (p<0.01) |
|  | Mean daily activity assessed by pedometer (arbitrary units per day) | | -1.2 (not significant) |
|  | Maximum exercise capacity (in Watt) | | -9 (not significant) |
| Casaburi 1997[41] | Increase in % of maximum exercise duration  with growth hormone and placebo | | 79% and 58% (not significant)  (no raw data available) |
| Casaburi 2004[37] | Endurance time (min) during constant work treadmill walk test | End of rehabilitation  (week 13):  At follow-up (week 25): | 5.35, p = 0.013  6.6, p = 0.009 |
| Casaburi 2004[24] | Maximum exercise capacity (in Watt) | | 4.5 (p > 0.05) |
|  | Exercise endurance (minutes at 80% of maximum exercise capacity) | | 3.6 (p > 0.05) |
|  | Muscle strength (in kg) | | 11 (p > 0.05) |
|  | Muscle fatigability (number of repetitions at 80% of one repetition maximum) | | 4 (p > 0.05) |
| Creutzberg  2003[36] (n_1_=28;n_2_=28) | SGRQ Total | | -6.4 (-13.2 to 0.4) |
|  | SGRQ Symptom | | -12.1 (-21.3 to –2.9) |
|  | SGRQ Activity | | -5.3 (-16.1 to 5.5) |
|  | SGRQ Impact | | -5.2 (-12.5 to 2.2) |
|  | Maximum exercise capacity  In all patients (in Watt)  In patients receiving maintenance treatment with oral glucocorticosteroids (in Watt) | | 5.4 (-2.4 to 13.2)  15.7 (7.8 to 23.6) |
| Gosselink 2001[38]  (n_1_=9;n_2_=9 for CRQ, n_1_=12;n_2_=12 for exercise tests) | CRQ total | | -0.05 (-0.67 to 0.57) |
|  | CRQ Dyspnea | | 0.40 (-0.48 to 1.28) |
|  | CRQ Fatigue | | 0.00 (-0.73 to 0.73) |
|  | CRQ Emotional function | | -0.57 (-1.37 to 0.23) |
|  | CRQ Mastery | | 0.25 (-0.70 to 1.20) |
|  | Functional exercise capacity (walking distance in meters) | | -6 (-74 to 62) |
|  | Maximum exercise capacity (in Watt) | | -1 (-13.4 to 11.4) |
| Steiner 2003[35]  (n_1_=25;n_2_=35) | CRQ Dyspnea | | -0.3 (-0.9 to 0.3) |
|  | CRQ Fatigue | | -0.1 (-0.5 to 0.3) |
|  | CRQ Emotional function | | 0.1 (-0.3 to 0.5) |
|  | CRQ Mastery | | -0.5 (-1.1 to 0.1) |
|  | Functional exercise capacity (walking distance in meters) | | 18 (–8 to 45). |
|  | Endurance shuttle walk test (seconds) | | 103 (–55 to 255). |

n1= Intervention group;n2= Control group, n3= Heliox group

CRQ, maximum and functional exercise capacity: Between group differences > 0 favors intervention group; HADS, SGRQ, LCADL Breathlessness at maximum exercise capacity (Borg scale): Between group differences > 0 favors control group
